# Application of infrared matrix-assisted laser desorption electrospray ionization mass spectrometry for morphine imaging in brain tissue

**DOI:** 10.1007/s00216-023-04861-x

**Published:** 2023-07-25

**Authors:** Yury Desyaterik, Joseph N. Mwangi, MaryPeace McRae, Austin M. Jones, Angela D. M. Kashuba, Elias P. Rosen

**Affiliations:** 1 Eshelman School of Pharmacy, University of North Carolina at Chapel Hill, Chapel Hill, NC USA; 2 Shattuck Labs, Durham, NC USA; 3 School of Medicine, University of Virginia, Charlottesville, VA USA; 4 School of Pharmacy, Virginia Commonwealth University, Richmond, VA USA

**Keywords:** Mass spectrometry imaging, MALDESI, Morphine

## Abstract

Here, we present a method developed for the analysis of spatial distributions of morphine in mouse brain tissue using infrared matrix-assisted laser desorption electrospray ionization (IR-MALDESI) coupled to a Q Exactive Plus mass spectrometer. The method is also capable of evaluating spatial distributions of the antiretroviral drug abacavir. To maximize sensitivity to morphine, we analyze various Orbitrap mass spectrometry acquisition modes utilizing signal abundance and frequency of detection as evaluation criteria. We demonstrate detection of morphine in mouse brain and establish that the selected ion monitoring mode provides 2.5 times higher sensitivity than the full-scan mode. We find that distributions of morphine and abacavir are highly correlated with the Pearson correlation coefficient *R* = 0.87. Calibration showed that instrument response is linear up to 40 pg/mm^2^ (3.8 μg/g of tissue).

**Graphical abstract:**

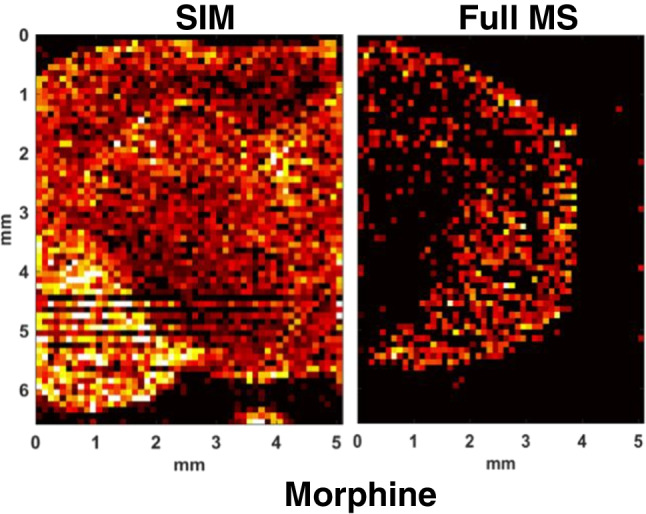

## Introduction

HIV infection spreads rapidly to the nervous system [1], where infected cells persist despite viral suppression in plasma by combination antiretroviral therapy thereby sustaining a viral reservoir in the brain [2]. The associated immune response can lead to neurocognitive and neurobehavioral impairment [3, 4]. The likelihood and severity of impairment increase among persons living with HIV who also have an opioid use disorder [5]. Morphine, for example, may alter permeability of the blood–brain barrier (BBB) and influence drug efflux transporter expression [6]. While there is some evidence to suggest that neurocognitive impairment may be associated with limited penetration of antiretrovirals (ARVs) into the brain [7], data on the spatial distributions of antiretrovirals (ARVs) within brain tissue are limited [8], as are any differences in ARV exposure driven by the additional presence of opioids.

Mass spectrometry imaging (MSI) offers an approach for label-free spatial mapping of multiple analytes simultaneously at high spatial resolution, providing the potential to investigate drugs and their metabolites while also characterizing endogenous metabolomic/lipidomic response from the same tissue sample. We have previously demonstrated infrared matrix-assisted laser desorption electrospray ionization (IR-MALDESI) as an MSI technique with high sensitivity for a range of ARVs in tissues, including brain tissue [8–12]. IR-MALDESI is well-suited to small molecule analysis because the sample ablation with infrared radiation can be performed using a simple layer of exogenous ice rather than high concentrations of an organic matrix. While MSI has been used to detect for the primary metabolite of heroin, 6-monoacetylmorphine [13], and opiate replacement therapies [14], it has not previously been used to evaluate morphine in tissue.

Analyte sensitivity can be a significant challenge for the targeted analysis of small molecules in the brain by mass spectrometry imaging. The BBB limits penetration of drug to the brain compartment such that concentrations of drug accumulating in brain tissue are low relative to plasma concentrations even for lipophilic compounds that are best suited for transit across the BBB. In addition, the volume of tissue sampled by MSI is inherently limited to preserve spatial resolution. Additionally, the lipid-rich endogenous ions that are generated during MSI analysis of brain tissue can lead to large tissue matrix effects that may result in peak interferences as well as ionization suppression of targeted analytes, reducing sensitivity. Strategies for improving sensitivity can be implemented throughout the analysis workflow. Sample washing techniques have been used to reduce lipid response in tissues, but these approaches can also delocalize small molecules. Ionization efficiency can be improved based on the chemical properties of the electrospray solvent to enhance sensitivity of tissue-derived neutral molecules or selectivity in ionization of certain compound classes [15]. A large burden of endogenous ions can also influence ion trapping and detection efficiency. Mass spectrometer acquisition modes that reduce the mass range of analyzed ions can also yield improved selectivity and sensitivity for targeted analytes.

Here, we develop a method for the analysis of morphine and abacavir in mouse brain tissue using IR-MALDESI. We compare multiple Orbitrap mass spectrometry acquisition modes to optimize sensitivity to morphine using signal abundance and frequency of detection as the criteria for assessment. We demonstrate detection of morphine in dosed mouse samples and investigate how morphine sensitivity influences its correlation with the spatial distribution of other measured ions.

## Materials and methods

### Materials and reagents

Methanol (HPLC grade), acetonitrile (HPLC), water (HPLC), and formic acid (Optima) were obtained from Fisher Scientific (Hampton, NH). Morphine standards were prepared from 1.0 mg/mL stock solution in methanol (Cerilliant®) ordered from Sigma-Aldrich.

### Animal study design

An animal study on adult female mice was performed under the approval of the Virginia Commonwealth University IACUC. Details of animal dosing have been described elsewhere [6]. Antiretrovirals were administered continuously for 5 days via an osmotic pump at doses calculated by allometric scaling (abacavir 2.5 mg/day). Morphine salt pentahydrate powder was diluted directly into the antiretroviral solution at a concentration of 2.0 mg/day.

### MSI analysis

Chemical standards and acquisition mode diagnostics were evaluated by direct electrospray injection into an Orbitrap mass spectrometer (ThermoFisher Q Exactive Plus, Bremen, Germany). The electrospray solvent was a mixture of water and either methanol or acetonitrile (50:50 v/v) with 0.2% formic acid. Analysis was conducted in positive mode, with resolving power set to 140,000 at *m/z* 200. The mass accuracy of better than 1 ppm was confirmed by weekly calibration of the mass spectrometer with the calibration solution recommended by the manufacturer. Acquisition mode was varied as part of our investigation. For MS/MS, a *m/z* 4 isolation window centered at *m/* × 286.10 was used with varying normalized collisional energy. In full-scan mode, the mass spectra were acquired with the mass range set to *m*/*z* 150–600. In selected ion monitoring (SIM) mode, a *m/z* 4 isolation window centered at *m*/*z* 286.10 was used. Automatic gain control (AGC) function was turned off to match trapping conditions required by IR-MALDESI MSI, and ion injection time was fixed at 11 ms. During direct injection, spectra were collected in each mode for 2 min (~ 200 scans).

Mouse brain samples were created by using a slicer matrix (Zivic Instruments, BSMAS001-1) to expose a posterior region of the brain and then sectioned coronally by clinical cryostat (Leica CM1950, Leica Biosystems, Nussloch, Germany) to a tissue thickness of 10 μm at approximately Bregma − 2. Tissue sections were thaw mounted on microscope slides and stored at − 86 °C until IR-MALDESI MSI analysis. Calibration of morphine response on brain tissue was performed by spiking 100 nL of standards (0.0, 0.016, 0.032, 0.063, 0.125, 0.25, 0.50, 1.0, and 2.0 μg/mL) directly onto mouse brain tissue sections collected in the same region from mice not dosed with drug. A calibration curve was created by exporting morphine signal abundance associated with each spot using MSiReader and then plotting the summed signal abundance over each calibration spot against the known spotted morphine concentration. To determine an absolute concentration, the slope and intercept of this curve were applied to the total voxel abundance value over the full area of the sample.

Conducting MSI analyses using an IR-MALDESI source has been described in detail elsewhere [9, 10, 16]. Briefly, prepared tissue sections were placed on a thermally controlled stage (a thermoelectric cooler TE Technology, Inc., Traverse City, MI) in the source chamber. Dry nitrogen was introduced to the closed chamber to reduce humidity, after which samples were cooled to − 9 °C. The source was then opened to ambient air, which increased the relative humidity to ~ 50% and allowed an ice film to form on the surface of the sample. The ice film was allowed to grow for approximately 10 min, corresponding to an ice film thickness of 100–150 μm (in a separate experiment, we estimated the thickness of the ice layer by weighing the sample slide before and after the ice growing procedure), after which the source was closed, and a low flow of nitrogen was re-introduced to maintain a relative humidity of ~ 15% during sample analysis, allowing for constant ice thickness. An IR OPO laser (Opotek, Carlsbad, CA) tuned to 2.94 μm was used for sample ablation. The plume of volatilized material from each sampling location was ionized by a perpendicular electrospray. Resulting ions were sampled into the Orbitrap QE + , which was triggered to start data acquisition simultaneously with the laser shot, and ion injection time was fixed to allow ion transition from the pulsed laser to the Orbitrap. Following acquisition, the sample stage was translated in 100-μm step increments. Laser, stage, and mass spectrometer signaling was controlled by a microcontroller and custom software [17].

MSConvert [18] was used to convert raw signal abundance data to.mzXML files, which were then loaded into the mass spectrometry imaging program MSiReader (v1.02, North Carolina State Univ., FTMS Lab.) to generate ion distribution maps [19] using default 5 ppm m*/z* tolerance window.

## Results and discussion

### Comparison of ion detection by MS acquisition mode by direct injection

**Fig. 1:**
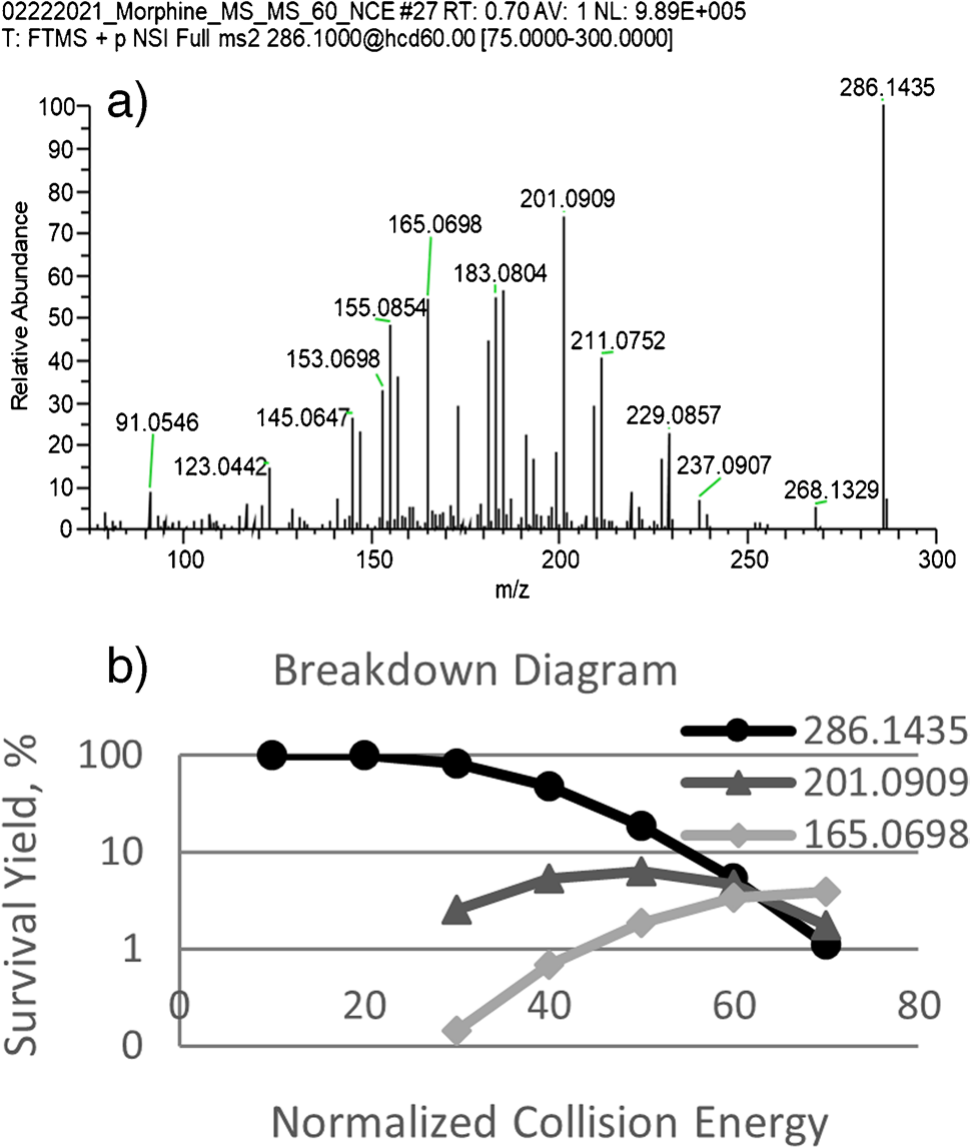
**a** MS/MS spectrum of morphine standard at normalized collision energy 60 eV, **b** breakdown diagram showing morphine precursor ion and two most abundant fragment peaks

Initial characterization of morphine was performed by direct infusion of a 1-μM standard in the electrospray solvent in three MS acquisition modes: (1) tandem MS; (2) full scan; and (3) SIM. Tandem MS is routinely used in LC–MS methods for opiate detection [20–22] including analysis of morphine [23–25] because of the added selectivity of the approach relative to full-scan MS analysis. A breakdown diagram showing proportional response of the morphine precursor peak (*m/z* 286.1435) and two fragments frequently used for morphine detection [21] (*m/z* 201.0909 and 165.0698) as a function of normalized collision energy applied in the Orbitrap HCD cell during tandem MS analysis can be seen in Fig. 1. The breakdown diagram shows that the characteristic morphine fragments reflect competing reaction channels, which ultimately divides the morphine response between smaller peaks. For example, the maximum signal abundance from morphine fragment *m/z* 201.0909, at normalized collision energy (NCE) 50%, is more than an order of magnitude lower than the signal abundance of the morphine precursor ion prior to fragmentation. A large number of additional fragments (ca. 20) from morphine are generated upon collisional activation, as shown in Fig. 1. These results indicate that tandem MS with high collisional energy should be avoided for detection of morphine in circumstances prioritizing high sensitivity.

**Fig. 2:**
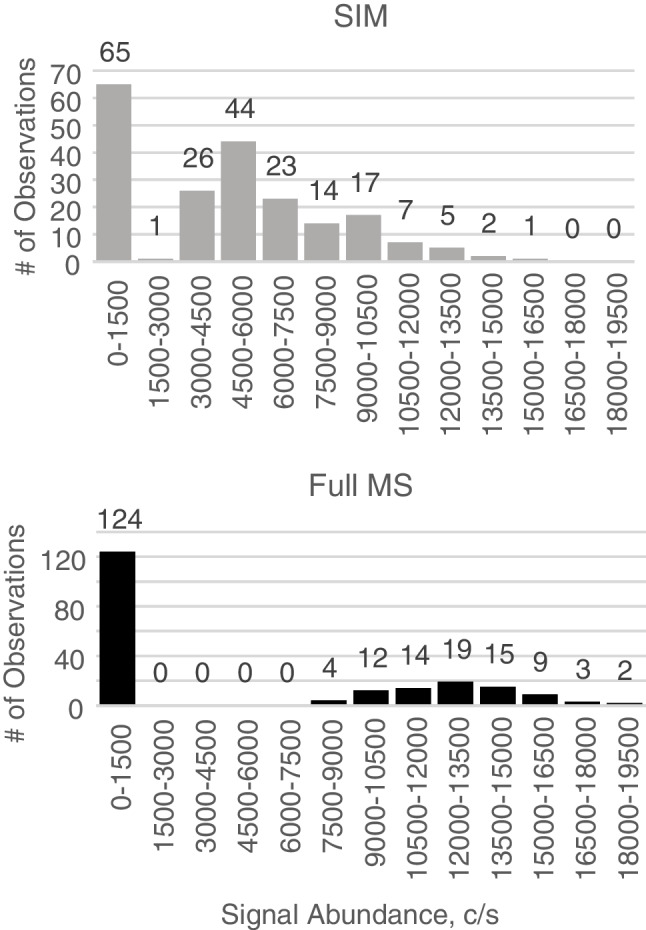
Histogram of ambient *m*/*z* 286.1732 peak signal abundance measurements in SIM (top) and full-scan (bottom) modes

We investigated detection frequency as a function of signal abundance and minimum peak height for a series of ambient ions analyzed along with the morphine standard to examine how the signal threshold varies for morphine detection between full-scan and SIM acquisition modes. Table 1 displays ratios of average signal abundance for selected ion peaks detected in both full-scan and SIM acquisition modes. Two values were calculated for each *m*/*z* value, utilizing two full MS (FMS) studies. For major ambient peaks that are detectable in every scan, signal abundance ratios between acquisition modes (SIM/FMS) vary between values greater than and less than 1 and do not indicate significant difference in sensitivity between the two acquisition modes (average ratio 1.14, SD 0.67). Examination of minor peaks (< 100% detection in collected scans) indicated that, although the average signal abundance was consistently higher in full-scan mode acquisition (average ratio 1.76, SD 0.40), frequency of detection was substantially higher in SIM mode (average ratio 4.32, SD 1.94). In order to understand the apparent discrepancy between average signal and detection frequency, we looked at the minimum reported peak abundance for the minor peaks in each mode, which were assumed to be close to the signal threshold. We discovered that the signal threshold in SIM mode is approximately two times lower than that in full-scan mode. A histogram of signal abundance values for a representative minor peak, *m*/*z* 286.1732, can be seen in Fig. 2 for SIM and full-scan modes. A comparison of these histograms demonstrates that the largest proportion of observations in SIM mode fall in a range of signal abundance (3000–7500) that is below the signal threshold in full-scan mode.

**Table 1:** Analysis of full MS and SIM modes using impurities in the electrospray. Ratios of average peak intensities in full MS and SIM modes, ratios of frequency of detection and minimal peak values in full MS and SIM modes. *FOD*, frequency of detection

Large peaks	*m/z*	286.3103	286.2820	286.2739	286.2456	286.2376	286.2012	286.1655
Abundance ratio	FMS/SIM	0.64 ± 0.02	0.65 ± 0	2.34 ± 0	0.8 ± 0.01	1.31 ± 0.05	1.86 ± 0.05	0.37 ± 0.01
Small peaks	*m/z*	286.1366	286.0132	286.1732	286.1827	287.0800	287.0870	287.1215
Abundance ratio	FMS/SIM	2.56 ± 0.05	1.42 ± 0.06	1.95 ± 0.03	1.29 ± 0.01	1.43 ± 0.04	1.81 ± 0.03	1.86 ± 0.02
FOD ratio	SIM/FMS	1.31 ± 0.06	6.64 ± 1.66	1.61 ± 0.14	4.93 ± 0.6	6.27 ± 0.11	4.58 ± 0.27	4.91 ± 0.17
Min. peak ratio	FMS/SIM	3.05 ± 0.1	2.75 ± 0.04	2.91 ± 0.09	2.9 ± 0.27	2.31 ± 0.09	2.69 ± 0.04	3.08 ± 0.02

SIM mode offers higher sensitivity than the full-scan mode in typical applications like LC–MS or direct sample injection via electrospray [26–28]. In the SIM mode, only ions from a narrow user-defined *m/z* window are transmitted into the mass spectrometer, as opposed to the full-scan MS mode, where the typical range is a few hundred to thousands of daltons. Since the total number of ions that can be analyzed in an ion trap mass spectrometer is limited by space charge effects, typically about a million ions in an Orbitrap [29], reducing the *m*/*z* window as in SIM mode leads to a drastic increase in the proportion of trapped ions associated with a targeted analyte and enhances sensitivity. Ordinarily, Orbitrap acquisition utilizes the automatic gain control (AGC) function to calculate trap fill time with a short pre-scan designed to control the number of ions in the trap. IR-MALDESI MSI using a pulsed IR laser requires the AGC function to be off, in order to ensure that ion injection time is sufficiently long (~ 11 ms) [30] for ions from the laser desorption event to be trapped. Here, we have demonstrated that even in the absence of AGC, the targeted *m*/*z* scan range provided by SIM offers 2–threefold higher sensitivity for ambient ions close to the limit of detection.

### Morphine analysis on mouse brain tissue

**Fig. 3:**
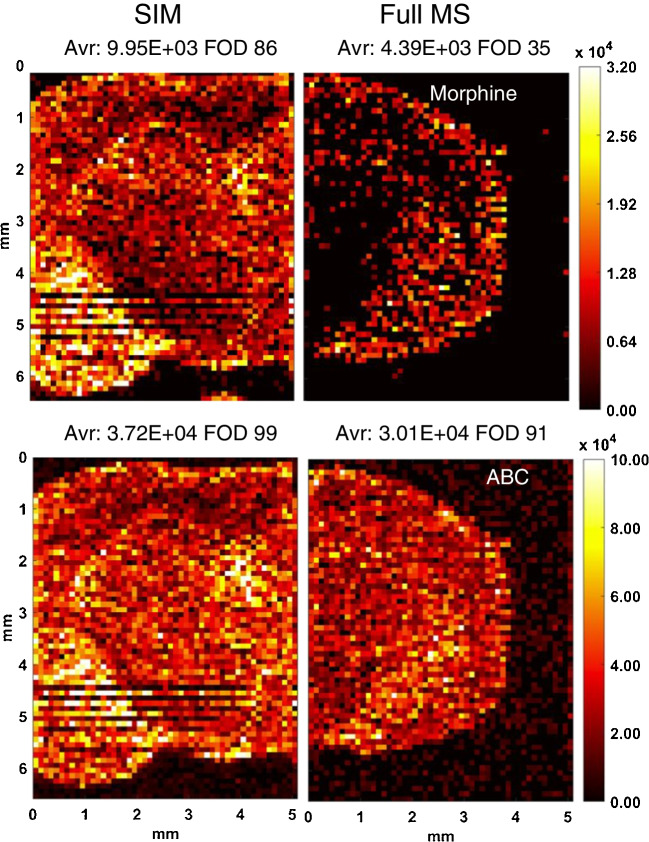
Comparison of ion maps of morphine and abacavir (ABC) collected in SIM and FMS modes. Hippocampus region. Average signal abundance and frequency of detection are shown on the top. Avr., average value of the signal abundance; FOD, frequency of detection %

Based on the observed differences in signal threshold between full-scan and SIM acquisition modes through direct infusion, we examined how sensitivity between these two acquisition modes differed for analysis of morphine and abacavir in mouse brain tissue. Concentrations of drug penetrating into mouse brain striatum and hippocampus, based on co-administering morphine and abacavir, are reported previously to be 99.9 ± 10.6 and 110.1 ± 11.5 ng/g abacavir and 259.0 ± 107.6 and 143.9 ± 31.1 ng/g morphine respectively [6]. Figure 3 shows ion maps of morphine and abacavir precursor ions collected by IR-MALDESI MSI analysis in both full-scan and SIM modes from the same tissue section of the hippocampus region of a mouse brain sample. Abacavir is detected throughout the tissue section in both acquisition modes at similar signal abundance. The frequency of detection for morphine is higher in SIM mode (86%) than in full-scan mode (35%), similar to the direct infusion observations.

**Fig. 4:**
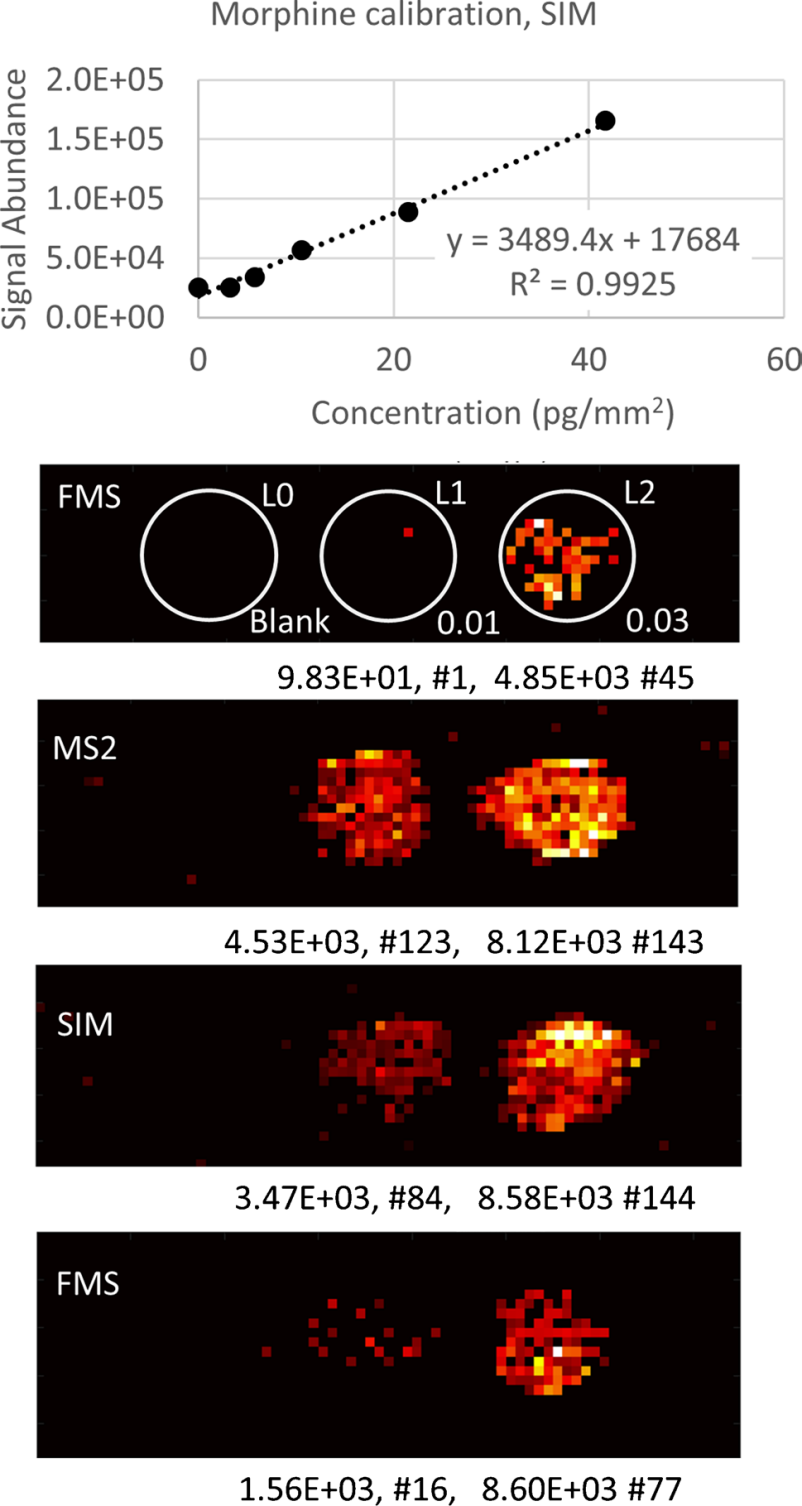
Morphine calibration curve for SIM mode, ion maps comparing morphine sensitivity in FMS, SIM, and MS2 modes. 100 nL of solution was deposited on the blank tissue. Concentrations of the calibration solutions (μg/mL) are shown on the figure. Average signal abundance and number of detected pixels are shown below each calibration spot

In order to assess the linear range of IR-MALDESI MSI response and limit of detection to morphine in mouse brain, we performed an on-tissue calibration by depositing 100 nL droplets of prepared morphine standards onto blank slices of mouse brain tissue. The morphine concentrations used in this initial experiment were 0.0 (blank), 0.063, 0.125, 0.25, 0.50, and 1.0 μg/mL (corresponding on-tissue concentrations 0.0, 3.2, 5.8, 10.5, 21.5, 41.7 pg/mm^2^), which were chosen to cover the range of relevant concentrations expected from analysis of dosed mouse samples. As shown in Fig. 4, the response is linear, with *R*^2^ = 0.99 over the selected range. The limit of detection (LOD) is routinely estimated based on the expression [31] LOD = 3*SD_blank_/Slope, where SD_blank_ refers to the standard deviation (SD) of the blank measurement and Slope is the slope of the linear interpolation of calibration data. One complication of this approach for data derived from an Orbitrap is that the detector noise level is not accessible and all signals below an internally calculated threshold level are algorithmically zeroed out. We can also estimate the signal abundance threshold using the value of the lowest peak in the acquired spectra, which for SIM mode is ~ 3000 c/s (counts per second). Assuming that the 3*SD of the noise is equal to the signal threshold, we estimate using slope from Fig. 4, LOD = 1 pg/mm^2^. Based on a tissue density of 1.046 g/cm^3^, within the range of observed densities for brain tissue [32], and a section thickness of 10 μm, this LOD reflects a concentration of 95 ng/g tissue. This hypothesis was tested by further diluting the lowest standard level of 0.063 μg/mL to 0.032 and 0.016 μg/mL, where the lowest concentration is equivalent to 0.8 pg/mm^2^ (75 ng/g tissue) in the deposited spot. Figure 4 demonstrates that while morphine is still detectable in SIM, and in a tandem MS mode with NCE = 10, it is undetectable in the full-scan acquisition mode. Figure 4 shows the average signal abundance and number of detected pixels. We repeated the full MS mode experiment because, at very low signal abundance, reproducibility is poor, as was observed by other groups [27, 33]. While this limit of detection is an estimate, it is consistent with the expected range of morphine tissue concentrations based on prior work and supports the higher frequency of detection of morphine from dosed samples in SIM mode relative to full-scan mode.

**Fig. 5:**
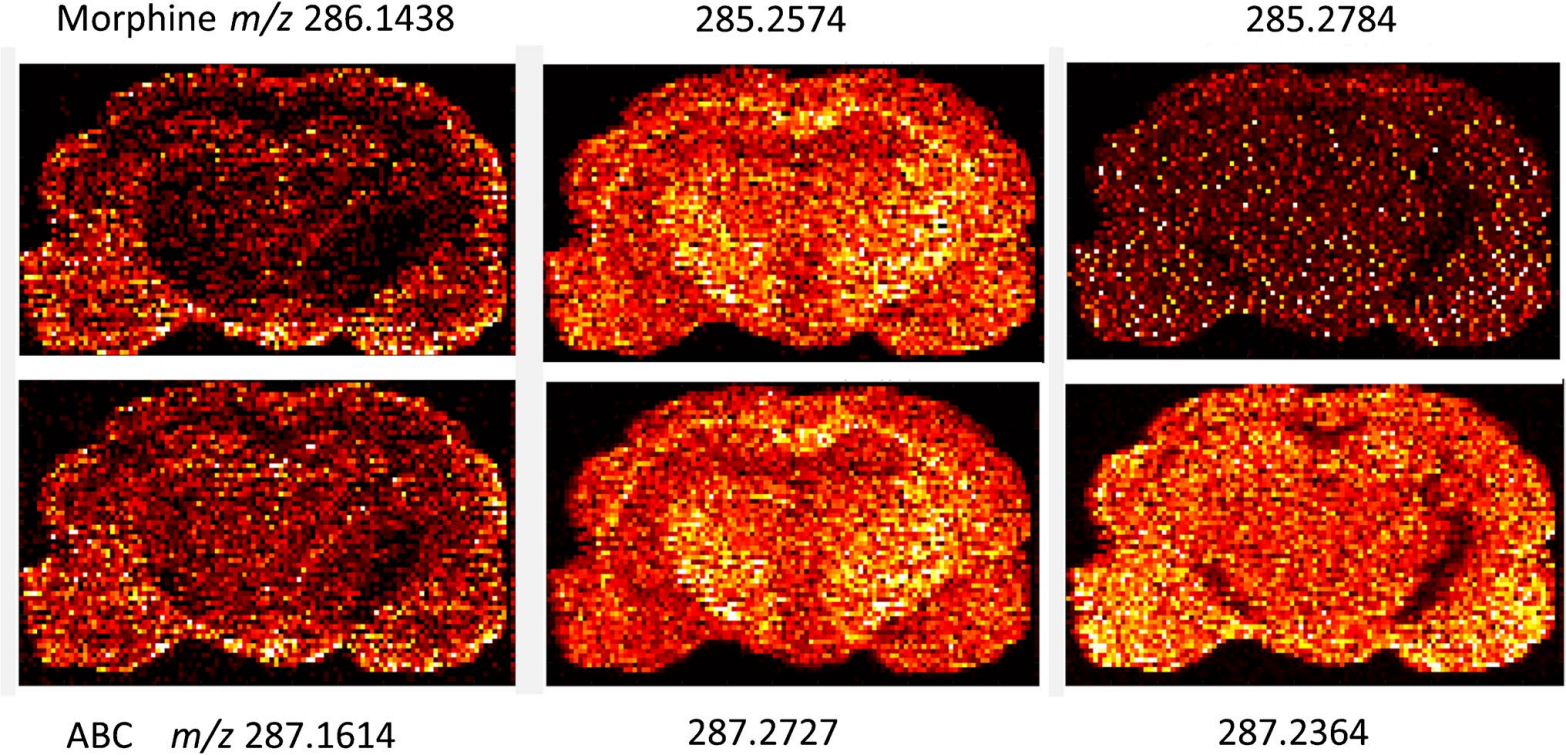
Ion maps for selected ions

**Fig. 6:**
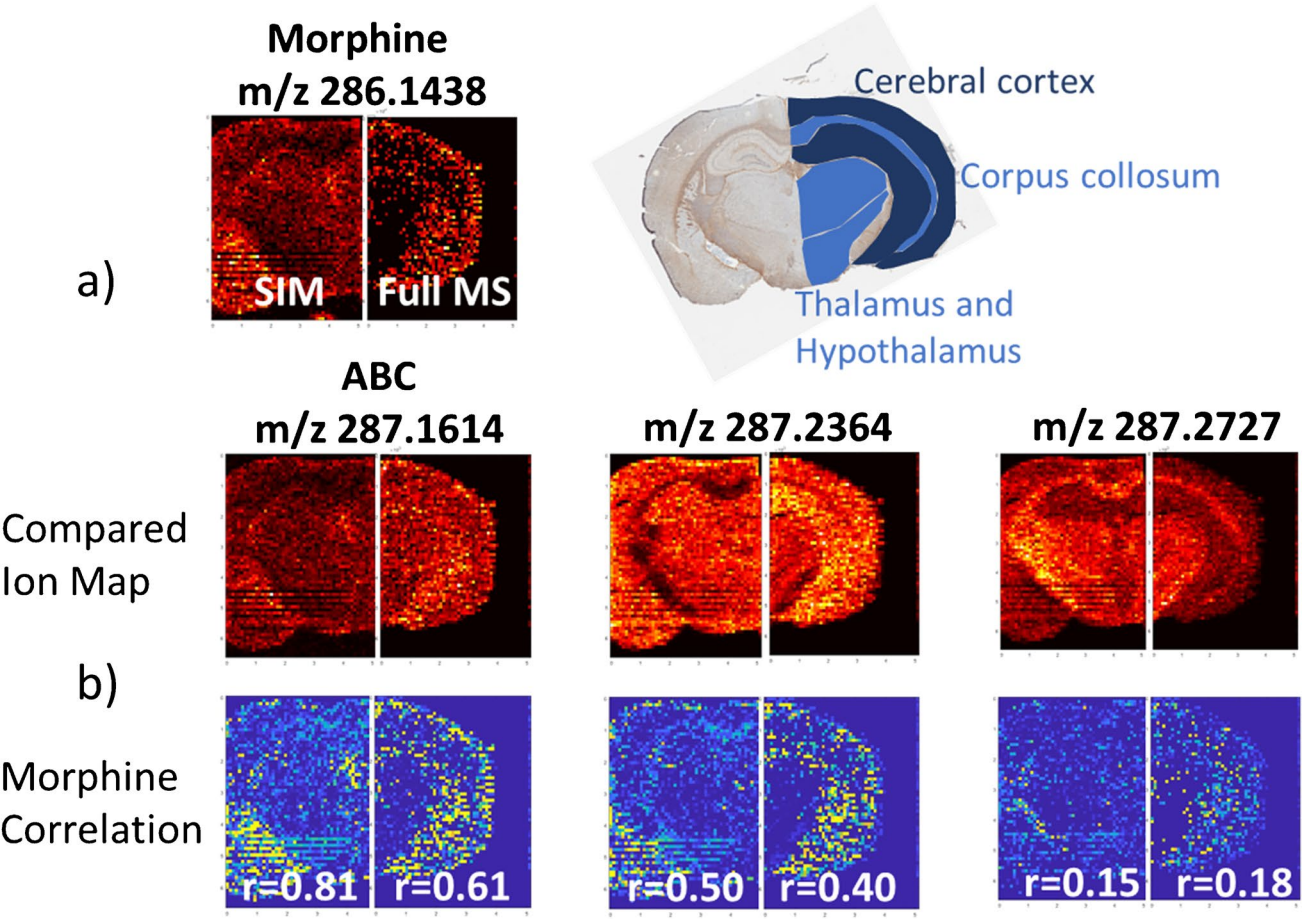
Correlation maps between morphine, abacavir, and two endogenous ions with differing regional signal abundance across the coronal section of posterior mouse brain in SIM (left) and full-scan (right) segments of each image. Relevant anatomical regions of this section are defined in the upper right labeled subjacent section

One of the primary goals of targeted pharmacological study of drugs by MSI is the spatial correlation of analyte distribution with microscopy imaging (e.g., histology or immunohistochemistry) or with other endogenous information derived from MSI analysis. Even with a narrow mass range for SIM acquisition targeting morphine and abacavir, we measured four endogenous ions that showed differing signal abundance across the coronal sections of the posterior brain, particularly in the region of the corpus callosum, thalamus, and hypothalamus (Fig. 5). We examined how the correlation mapping between morphine and these other ions differs between SIM acquisition and the lower sensitivity full-scan method. The enhanced frequency of morphine detection by SIM in the posterior brain is particularly evident relative to full scan in the central brain stem region of the thalamus and hypothalamus, shown in Fig. 6a. Correlation maps between morphine and abacavir or two ions reflecting the different observed regional accumulation can be seen in Fig. 6b. The higher frequency of morphine detection on-tissue by SIM mode results in higher composite correlation coefficients relative to full-scan mode when comparing morphine to ions measured across the different morphological regions in similar relative abundances (abacavir and the endogenous ion *m*/*z* 287.2364). For an ion (*m*/*z* 287.2727) with higher local signal abundance in the corpus callosum, thalamus, and hypothalamus reflecting a very different pattern of distribution than morphine, there was not an apparent difference in correlation coefficients between SIM and full-scan modes. Pearson correlation coefficients comparing the morphine distribution with other ions can be seen in Table 2. Overall, the quality of information provided by correlation mapping is improved when the reference distribution has a high frequency of detection, which has relevance for evaluating theories about the variables influencing the drug biodistribution. The higher frequency of detection of morphine provided by SIM mode is also important when correlating its spatial distribution to the distribution of other endogenous ions or anatomical information from histology. Our assessment of ion correlation mapping has focused on prominent anatomical features identifiable from posterior brain tissue sections collected at an estimated Bregma − 2.0 mm in order to describe how changes in analyte sensitivity influence analyses of covariance. Our primary motivation was to demonstrate the capability of measuring morphine throughout a brain cross section and we have not sought to perform an exhaustive microdissection of morphine penetration into all existing anatomical regions present, which we acknowledge may not be possible for very small features using this method given our spatial resolution of 100 mm. Methods of image fusion [34], which utilize high-resolution optical microscopy to inform the interpolation of MSI data, may be required for applications where assessment of morphine penetration into such regions is needed.

**Table 2:** Pearson correlation coefficient of abundance of the selected ions and morphine for data from Fig. 5. *m/z* 286.1438 morphine, 287.1614 abacavir

*m/z*	285.2574	285.2784	286.1438	287.1614	287.2364	287.2727
*R*	0.09	0.03	1.00	0.87	0.40	0.05

## Conclusions

In this work, we demonstrated a method for measuring morphine distribution in mouse brain tissue by IR-MALDESI MSI. We show that tandem MS has approximately an order of magnitude lower sensitivity than measurement of the morphine molecular ion due to multiple parallel fragmentation pathways. Even without the use of automatic gain control, reducing the operating scan range through the use of selected ion monitoring considerably increases the frequency of peak detection for low abundance peaks, increasing sensitivity by ~ 2.5-fold. Using this approach, we were able to detect morphine standards on mouse brain tissue in concentrations as low as 0.8 pg/mm^2^. The increased frequency of detection for morphine provided by this method improves the quality of correlation analysis between morphine spatial distribution and other endogenous ions evaluated simultaneously and will facilitate multimodal imaging assessment of factors influencing its biodistribution.

Finally, we discovered that there is a substantial correlation between the distribution of morphine and that of abacavir, which can be helpful in identifying factors that influence its biodistribution.
